# Shoulder Injury Related to Vaccine Administration (SIRVA) After COVID-19 Vaccination: A Retrospective Study

**DOI:** 10.7759/cureus.98023

**Published:** 2025-11-28

**Authors:** Gopikanthan Manoharan, Thivagar Murugesan, Jo Winton, Matthew Smith, Peter Brownson

**Affiliations:** 1 Orthopaedics and Trauma, Royal Shrewsbury Hospital, Shrewsbury, GBR; 2 Trauma and Orthopaedics, Robert Jones and Agnes Hunt Hospital, Oswestry, GBR; 3 Orthopaedic Surgery, Shrewsbury and Telford NHS Foundation Trust, Shrewsbury, GBR; 4 Upper Limb Clinical Physiotherapy, Liverpool University Hospital NHS Foundation Trust, Liverpool, GBR; 5 Trauma and Orthopaedics, Liverpool University Hospital NHS Foundation Trust, Liverpool, GBR

**Keywords:** adverse event, covid-19, shoulder injury related to vaccine administration, sirva, subdeltoid bursitis, vaccination, vaccine hesitancy

## Abstract

Background: The global administration of billions of COVID-19 vaccine doses has raised concerns about potential adverse effects, contributing to vaccine hesitancy. While transient mild discomfort is common after intramuscular vaccination, persistent and severe post-vaccination shoulder pain has led to recognition of Shoulder Injury Related to Vaccine Administration (SIRVA). SIRVA is characterised by shoulder pain and restricted range of motion typically occurring within 48 hours of inoculation, thought to result from inadvertent vaccine delivery into the subdeltoid bursa. In addition to its clinical definition, SIRVA is also viewed as a medicolegal construct, particularly in the context of vaccine injury compensation frameworks. This study aimed to describe our experience with patients presenting with SIRVA-like symptoms following COVID-19 vaccination, compare these findings with published literature, and assess clinical outcomes.

Methods: A retrospective study was conducted at a major trauma centre. All patients presenting to the orthopaedics department with atypical shoulder symptoms following COVID-19 vaccination between January and December 2021, with at least six months of follow-up, were reviewed. Only patients meeting the Health Resources and Services Administration (HRSA) Vaccine Injury Table diagnostic criteria for SIRVA were included. Data on demographics, vaccine type, clinical findings, investigations, treatment, and outcomes were collected.

Results: Of the 31 patients presenting with post-vaccination shoulder symptoms, 16 (52%) met the HRSA criteria for SIRVA. The mean age was 54 years, and 63% were female. All patients presented with shoulder pain and reduced range of motion. The mean follow-up duration was 12 months. Most patients (94%) were treated non-operatively with analgesia, nonsteroidal anti-inflammatory drugs (NSAIDs), and physiotherapy. At one-year follow-up, 44% achieved complete or near-complete recovery, while 31% (n = 5) reported no improvement and required specialist referral.

Conclusion: SIRVA is a rare complication following COVID-19 vaccination and is best considered a medicolegal term rather than a definitive diagnosis. Strict adherence to diagnostic criteria is essential, as overdiagnosis may occur among patients with coincidental post-vaccination shoulder pain. While most cases resolve with conservative management, a subset may experience persistent symptoms. Evidence suggests that SIRVA is more likely related to improper injection technique rather than the vaccine itself.

## Introduction

On March 11, 2020, the World Health Organization (WHO) declared coronavirus disease 2019 (COVID-19) a pandemic [1]. Within the following 24 months, 62.5% of the global population received at least one dose of a COVID-19 vaccine, and 10.6 billion doses were administered worldwide [2]. In the United Kingdom (UK) and the United States of America (USA), 77% and 76% of the populations, respectively, had been vaccinated within two years; however, 19% of the UK population and around 25% of the US population remained unvaccinated and unwilling to receive a vaccine [2].

In 2019, the WHO identified vaccine hesitancy as one of the top ten global health threats [3]. A European survey published in 2020 found that 55% of respondents unwilling to receive the COVID-19 vaccine were concerned about potential side effects [4]. A common concern was that COVID-19 vaccines were experimental and lacked sufficient safety data. The UPTAKE study, published in 2021 and based on the UK population, reported that 6.8% of respondents would not receive a COVID-19 vaccine, with the most common reason being fear of side effects [5].

Transient mild discomfort is a well-known and commonly reported side effect of intramuscular vaccination into the deltoid muscle. However, there have been reports of more persistent and severe shoulder pain following vaccination [6]. Hibbs et al. reported that the average incidence of atypical shoulder pain and dysfunction following influenza vaccination, based on the Vaccine Adverse Event Reporting System (VAERS), was approximately 2% over a seven-year period [7].

The term SIRVA (Shoulder Injury Related to Vaccine Administration) was introduced by Atanasoff et al. in 2010 to describe such presentations [8]. SIRVA is characterised by shoulder pain and restricted range of motion typically occurring within 48 hours of intramuscular vaccine administration in the deltoid region [9]. However, diagnostic criteria are heterogeneous internationally, with the HRSA description reflecting a medicolegal classification framework used primarily in the United States rather than an internationally standardised clinical definition.

The Vaccine Injury Table, published by the Health Resources and Services Administration (HRSA) of the USA, outlines the diagnostic criteria for SIRVA, including onset of shoulder pain within 48 hours of vaccination, restricted range of motion, and absence of prior shoulder pathology [10]. The pathophysiology of SIRVA remains poorly understood. Some authors suggest that the condition results from inadvertent injection of the vaccine through the deltoid muscle into the subdeltoid bursa, triggering an acute inflammatory response and prolonged symptoms [9,11]. This makes SIRVA a potentially avoidable complication. Based on this evidence, the US federal Vaccine Injury Compensation Program (VICP) removed SIRVA from its compensation table, citing "nearly uniform agreement in the scientific community that SIRVA is caused by improper vaccine administration rather than by the vaccine itself" [12]. However, most existing literature is based on small case series and observational studies, and a definitive causal relationship between vaccination and SIRVA cannot be conclusively established [13].

The reported incidence of SIRVA appears to be increasing, along with associated injury claims [14]. This may reflect growing public awareness of SIRVA as a compensable injury rather than an actual rise in incidence [7].

The aim of this study was to report our experience of patients presenting with SIRVA-like symptoms following COVID-19 vaccination and to compare our findings with those reported in the literature.

## Materials and methods

This retrospective observational study was conducted at a major trauma centre in the United Kingdom. Formal institutional review board (IRB) approval was not required, as the study involved a retrospective review of anonymised patient data with no direct patient contact or intervention. The study was conducted in accordance with the principles of the Declaration of Helsinki, and patient consent for use of anonymised data for research and publication purposes was obtained in accordance with institutional policy.

Patients presenting with new-onset shoulder pain or dysfunction following COVID-19 vaccination between January 2021 and December 2021 were identified using the Patient Episode Notification System (PENS) and the Picture Archiving and Communication System (PACS). The search name used included Shoulder, Shoulder pain, and vaccine.

Patients were included if they developed shoulder pain and/or restricted range of motion within 48 hours of receiving an intramuscular COVID-19 vaccine in the deltoid region, had a minimum follow-up of six months, and had sufficient documentation to assess the diagnostic criteria for SIRVA as defined by the Health Resources and Services Administration (HRSA) Vaccine Injury Table [10].

Patients were excluded if their medical records were incomplete, if they had pre-existing shoulder conditions such as rotator cuff tears or arthritis, if their shoulder symptoms followed injections at sites other than the deltoid, or if symptom onset occurred more than 48 hours after vaccination. Previous records were used to exclude pre-existing pathology.

Data were collected retrospectively using a standardised proforma and included patient demographics (age and sex), vaccination details (type, manufacturer, dose number, and date of administration), clinical features (pain location and severity, range of motion limitation, rotator cuff strength, and sensory deficits), investigations (including imaging modality and key findings), and treatment and outcomes (analgesia, NSAIDs, physiotherapy, corticosteroid injection, hydrodilatation, and degree of symptom resolution).

Patients were assessed by orthopaedic or musculoskeletal physiotherapy teams. ROM was measured using a goniometer where recorded, and functional recovery was evaluated through clinical documentation of pain relief, mobility, and daily activity performance. No validated functional outcome scoring systems (e.g., the Visual Analogue Scale, Constant-Murley Score) or proprietary tools were used. Follow-up assessments were typically conducted at six weeks, three months, and six months, with outcomes recorded at the final documented visit. Radiographs were performed to exclude structural pathology, while ultrasound was used selectively to evaluate bursitis or tendinopathy. MRI was reserved for atypical or persistent presentations.

All data were entered into Microsoft Excel and analysed descriptively. Continuous variables were summarised as means with ranges, and categorical variables as frequencies and percentages. Due to the small sample size, no inferential statistical analyses were performed.

No assessment scales, scoring systems, or proprietary tools were used in this study. The diagnostic evaluation was based entirely on the SIRVA criteria as defined in the Vaccine Injury Table published by the U.S. Health Resources and Services Administration (HRSA) [10]. These criteria are publicly available in the U.S. Federal Register and do not require permission or licensing for use in research or publication.

## Results

A total of 31 patients presented with atypical shoulder symptoms following COVID-19 vaccination. Five patients were excluded due to incomplete symptom onset data (n = 4) or inadequate follow-up (n = 1, four months). Ten further patients were excluded for not meeting SIRVA criteria, with symptom onset greater than 48 hours to one week (n = 5) or greater than one week (n = 5). Sixteen patients who met the SIRVA diagnostic criteria given by the HRSA were included in this study. Table 1 explains the SIRVA diagnostic criteria based on the HRSA Vaccine Injury Table [10].

**Table 1:** SIRVA diagnostic criteria based on the HRSA Vaccine Injury Table [10] HRSA: Health Resources and Services Administration.

S. No	Diagnostic Criteria
1	No history of pain, inflammation, or dysfunction of the affected shoulder prior to intramuscular vaccine administration that would explain the alleged signs, symptoms, examination findings, and/or diagnostic studies occurring after vaccine injection
2	Pain occurs within the specified time frame: 48 hours
3	Pain and reduced range of motion are limited to the shoulder in which the intramuscular vaccine was administered
4	No other condition or abnormality is present that would explain the patient's symptoms (e.g., NCS/EMG or clinical evidence of radiculopathy, brachial neuritis, mono-neuropathies, or any other neuropathy)

The mean age of SIRVA patients was 54 years, with more females (n = 10) than males (n = 6). The mean follow-up duration was 12 months (range: 6-14 months). The AstraZeneca vaccine was the most frequently associated, and most cases occurred after the first or second dose. Table 2 explains the study demographics of patients.

**Table 2:** Demographics AZ: Oxford AstraZeneca, PZ: Pfizer BionTech, COPD: chronic obstructive pulmonary disease.

Case Number	Sex	Age	Vaccine/Dose	Past Medical History and Allergies	Previous Shoulder Pathology
1	F	66	AZ/1	Shellfish allergy	Nil
2	M	31	AZ/1	Cyclizine allergy	Nil
3	F	59	PZ/2	Diabetes, hyperthyroidism, spina bifida	Nil
4	F	36	AZ/1	Migraine, psychiatric ailments	Nil
5	M	56	AZ/1	Nil	Nil
6	M	55	AZ/1	Diabetes	Nil
7	M	71	AZ/2	Diabetes, atrial fibrillation, lung cancer	Nil
8	M	56	AZ/1	Vitamin B12 deficiency, vitamin D deficiency	Nil
9	F	67	AZ/1	Allergic rhinitis, trigeminal neuralgia	Nil
10	F	50	PZ/3	Viral meningitis	Nil
11	F	57	AZ/2	Hypothyroidism, hypertension, migraine	Nil
12	F	50	PZ/2	Nil	Rotator cuff repair surgery, ipsilateral
13	F	50	AZ/1	Nil	Nil
14	M	51	AZ/2	Nil	Nil
15	F	53	PZ/3	COPD, Crohn’s disease, epilepsy	Nil
16	F	62	AZ/2	Diabetes, hyperthyroidism	Nil

At initial presentation, ROM was limited due to pain: mean flexion was 116° (range: 40-180°), and mean abduction was 106° (range: 30-180°). External rotation was <30° in 44% (n = 7), ~50° in 25% (n = 4), and unaffected in 31% (n = 5). Internal rotation (hand behind back) reached the thoracic or lumbar spine in 44% (n = 7) and only the gluteal region or greater trochanter in 50% (n = 8). Rotator cuff strength was reduced in 38% (n = 6). ROM improved with analgesia, reassurance, and physiotherapy. Two patients developed frozen shoulder. Table 3 explains the clinical findings of patients in this study.

**Table 3:** Clinical findings GT: greater tuberosity, US: ultrasound, MRI: magnetic resonance imaging, ACJ OA: acromioclavicular joint osteoarthritis, NAD: no abnormality detected.

Case No.	Symptom Onset	Symptoms	Range of Motion (ROM)	Cuff Power	Sensory Deficit Around the Shoulder	Imaging
1	24 hours	Shoulder pain	Normal	Reduced	Nil	None
2	Immediate	Shoulder pain, radiation down the arm	Reduced	Reduced	Present	X-ray - NAD
3	24 hours	Shoulder pain, radiation down the arm, stiffness	Reduced	Normal	Present	X-ray – NAD, MRI – mild bursal thickening
4	24 hours	Shoulder pain, radiation down the arm	Normal	Normal	Nil	None
5	2 hours	Shoulder pain, radiation down the arm	Reduced	Reduced	Nil	None
6	Immediate	Shoulder pain, radiation down the arm	Reduced	Reduced	Nil	None
7	48 hours	Shoulder pain, radiation down the arm	Reduced	Reduced	Nil	X-ray – ACJ OA
8	48 hours	Shoulder pain, radiation down the arm	Reduced	Reduced	Nil	X-ray - NAD
9	Immediate	Shoulder pain	Reduced	Reduced	Nil	None
10	Immediate	Shoulder pain, radiation down the arm	Normal	Reduced	Nil	None
11	Immediate	Shoulder pain	Normal	Reduced	Nil	X-ray - NAD
12	24 hours	Neck pain	Normal	Normal	Present	None
13	48 hours	Shoulder pain	Normal	Normal	Nil	None
14	24 hours	Stiffness	Reduced	Reduced	Nil	X-ray – GT sclerosis
15	24 hours	Shoulder pain, stiffness	Reduced	Normal	Nil	X-ray – GT sclerosis, ACJ OA
16	48 hours	Shoulder pain	Normal	Normal	Nil	US – thick bursa

Plain radiographs were performed in 44% (n = 7/16) and revealed greater tuberosity sclerosis in 12.5% (n = 2/16) and acromioclavicular joint arthritis in 6% (n = 1/16). Ultrasound was performed in one patient (6%) and showed bursal thickening. One patient underwent MRI, which showed mild bursal thickening.

Fifteen patients (94%) received analgesia, NSAIDs, and physiotherapy; only one did not receive physiotherapy. Among those treated, additional interventions included corticosteroid injection (n = 3), hydrodilatation (n = 1), or acupuncture (n = 1). None required surgery. Table 4 explains the treatment and outcomes of the patients in this study.

**Table 4:** Treatment and outcomes DoV: date of vaccine, NSAIDs: non-steroidal anti-inflammatory drugs, N/A: not applicable.

Case Number	Follow-up (Months)	Treatment	Current Status (Symptoms Resolved)	Time to Complete Resolution From DoV (Months)	Comments About Injection Site/Technique
1	12	Analgesia/NSAIDs, physiotherapy, corticosteroid injection	Not at all	N/A	Needle pointing upward
2	14	Analgesia/NSAIDs, physiotherapy, acupuncture	Partially	N/A	Injection too high
3	13	Analgesia/NSAIDs, physiotherapy	Nearly 100%	9	Nil
4	13	Analgesia/NSAIDs, physiotherapy	Not at all	N/A	Nil
5	14	Analgesia/NSAIDs, physiotherapy	Mostly	N/A	Nil
6	14	Analgesia/NSAIDs, physiotherapy	Not at all	N/A	Nil
7	12	Analgesia/NSAIDs, physiotherapy	Partially	N/A	Nil
8	12	Analgesia/NSAIDs, physiotherapy, corticosteroid injection	Not at all	N/A	Nil
9	12	Analgesia/NSAIDs, physiotherapy	Mostly	N/A	Nil
10	6	Analgesia/NSAIDs, physiotherapy	Partially	N/A	Nil
11	11	Analgesia/NSAIDs, physiotherapy	Partially	N/A	Nil
12	13	Analgesia/NSAIDs	Not at all	N/A	Nil
13	14	Analgesia/NSAIDs, physiotherapy	Complete	12	Nil
14	11	Analgesia/NSAIDs, physiotherapy, corticosteroid injection	Complete	7	Nil
15	7	Analgesia/NSAIDs, physiotherapy, hydrodilatation	Complete	7	Nil
16	14	Analgesia/NSAIDs, physiotherapy	Mostly	N/A	Nil

Four patients (25%) achieved complete symptom resolution at a mean of 11.3 months post-vaccination. Symptoms mostly resolved in three others (19%) at the same mean interval. Five patients (31%) had no improvement after 12.3 months. Two of these received corticosteroid injections and were referred for specialist review, and one was later referred for physiotherapy. One patient reported that the injection site seemed "too high," but most could not recall the injection technique.

## Discussion

This study reports 16 cases of SIRVA following COVID-19 vaccination in one of the largest single-centre series from the UK. Only 62% (n = 16/26) of patients presenting with shoulder pain after vaccination met HRSA diagnostic criteria [10]. A systematic review by Fortier et al. [15] identified 22 studies comprising 81 patients with post-COVID-19 vaccination SIRVA, of whom only 49% (n = 40/81) clearly met the HRSA criteria [10]. Fifty-three per cent of these cases were reported from Asia.

These data suggest that true SIRVA incidence is very low, given the vast global vaccination numbers, and that overdiagnosis may occur due to limited awareness of diagnostic criteria.

Risk factors

Known risk factors for SIRVA include extremes of BMI, older age (mean approximately 50 years), and female sex [7,8,14,16,17]. BMI was not recorded in this cohort. The mean age in our study was 54 years (vs 58 years in Fortier et al.'s study), with 63% female patients (vs 68% in Fortier et al.'s study). There is no conclusive evidence linking needle length or injection site with SIRVA development, although improper technique is suspected.

Clinical presentation

Eighty-eight percent of patients in our cohort reported shoulder pain, and 63% had reduced range of motion (ROM). Thirty-eight percent presented with both. Rotator cuff weakness was observed in 63%. These findings are comparable to those of Fortier et al.'s study, where 100% of patients reported pain and 87.7% had reduced ROM.

Investigations

In Fortier et al.’s review, 55.6% underwent MRI, 28.4% ultrasound, 25.9% X-ray, and smaller numbers underwent CT. Diagnoses included subacromial/subdeltoid bursitis (32%), adhesive capsulitis (27%), tendinopathy or cuff tear (21%), and Polymyalgia Rheumatica-like syndromes (17%) [15]. In contrast, 46% of our patients received no imaging. MRI can identify inflammatory changes such as bursitis and tendinitis and is recommended where available, although ultrasound may serve as a cost-effective alternative.

Treatment

In Fortier et al., treatment included physical therapy (35%), NSAIDs (33%), oral steroids (25%), corticosteroid injections (21%), and surgery (2.5%). In our study, physiotherapy and analgesia formed the mainstay (94%), with few requiring additional corticosteroid injections or hydrodilatation. No patients required surgery.

Outcomes

At 12 months, 44% of our patients achieved near-complete to complete recovery, while 31% had persistent symptoms. This compares to Fortier et al., where 52% achieved full recovery within eight months and 5.5% had persistent symptoms. Most patients recover with conservative management, although recovery times vary and can exceed eight months.

Current guidance

The UK Health Security Agency (UKHSA), referencing the Green Book, recommends using a 25 mm needle for COVID-19 vaccine administration, increasing to 38 mm in patients with greater adipose tissue [18-20]. The vastus lateralis muscle may be used where deltoid insufficiency is a concern. Similar recommendations exist in the CDC and Australian Immunisation Handbook guidelines [21,22].

## Conclusions

SIRVA is often discussed in medicolegal contexts rather than as a well-defined clinical diagnosis, and its pathophysiology remains uncertain. While the HRSA criteria aid recognition, the overall incidence is very low relative to global vaccination rates. It is plausible that many cases represent improper injection technique rather than a vaccine-specific complication. Nevertheless, new-onset shoulder pain is common, and temporal association alone does not confirm causation.

Most patients can be managed non-operatively, with good recovery through analgesia and physiotherapy, although some may experience prolonged symptoms. The clinical features, management, and outcomes of SIRVA after COVID-19 vaccination appear similar to those following other vaccines. Further research is warranted to clarify whether SIRVA represents a distinct clinical entity or an avoidable injection-related injury. Adherence to UK Green Book vaccination technique guidelines remains the best preventive measure.
